# Analysis of risk factors of precocious puberty in children

**DOI:** 10.1186/s12887-023-04265-x

**Published:** 2023-09-11

**Authors:** Yan Dong, Lili Dai, Yang Dong, Na Wang, Jing Zhang, Chao Liu, Zhifang Li, Limin Chu, Sisi Chen

**Affiliations:** 1 Department of Pediatrics, The First Hospital of Hebei Medical University, No. 89 Donggang Road, Yuhua District, Shijiazhuang, 050000 Hebei China; 2 Department of Laboratory Medicine, The First Hospital of Hebei Medical University, Shijiazhuang, China; 3 Department of Laboratory Medicine, The Third Hospital of Shijiazhuang, Shijiazhuang, China

**Keywords:** Children, Risk factors, Precocious puberty

## Abstract

**Background:**

The purpose of this study is to explore the related factors of precocious puberty in children.

**Methods:**

1239 children who underwent physical examination in our hospital from January 2020 to December 2022 were analyzed, including 198 precocious children and 1041 normal children. According to the age of 198 precocious children and 1041 normal children, 205 normal children were selected, and the remaining 836 normal children were excluded. They were divided into precocious group and normal group. The general data of the two groups were recorded. Logistic regression was used to analyze the influencing factors of precocious puberty in children.

**Results:**

There were statistically significant differences (P < 0.05) between the two groups in sex, bone age, daily exercise time, E2, FSH, LH, leptin, mother’s menarche time, living environment, consumption of nutritional supplements, consumption of foods containing pigments and preservatives, consumption of high-protein foods, and sleeping time. The multifactor logistic regression analysis shows that the risk factors of children’s precocious puberty included gender (female), bone age (> 10 years old), and daily exercise time (< 0.9 h), E2 (≥ 66.00pmol/L), FSH (≥ 6.00U/L), LH (≥ 3.50U/L), leptin (≥ 8.00 µ G/L), mother’s menarche time (< 12 years old), living environment (chemical industry zone), consumption of nutritional supplements (often), consumption of high-protein food (often), and sleep time (< 10 h).

**Conclusion:**

In conclusion, children’s gender, bone age, exercise habits, E2, FSH, LH, leptin, mother’s menarche time, living environment, eating habits, sleep time and other factors are closely related to precocious puberty in children. Reminding parents to actively prevent related factors in clinical work is helpful to prevent the occurrence of precocious puberty in children.

## Introduction

Precocious puberty refers to the early emergence of secondary sexual characteristics in children, with girls having secondary sexual characteristics before the age of 8 or menstruation before the age of 10, and boys having secondary sexual characteristics before the age of 9. The main clinical manifestations of girls’ secondary sexual characteristics are breast development, pubic hair and armpit hair growth, pelvic widening, while the main manifestations of boys’ secondary sexual characteristics are testicular enlargement, penis enlargement, development of armpit hair, seminal vesicle and prostate [1, 2]. According to pathogenesis and clinical manifestations, precocious puberty can be divided into central (gonadotropin-releasing hormone dependent) and peripheral (non-gonadotropin-releasing hormone dependent) precocious puberty [3]. In recent years, studies have pointed out that the incidence of precocious puberty in children is increasing year by year, and the trend is younger [3]. Precocious puberty in children will lead to increased hormone secretion in children, which will affect their psychology, height, nervous system tumors or common hypothalamic hamartoma and pituitary adenoma, etc., seriously endangering children’s physical and mental health, and may also advance the time of bone maturity in children, resulting in short adult height. And may increase the risk of high blood pressure, diabetes, obesity, and infertility in adulthood [4]. Previous studies showed [5–7] that the occurrence of early puberty in children may be related to gender, leptin, daily activity time, etc., but the results of each study are not completely consistent. Therefore, it is necessary to identify the relevant factors that cause precocious puberty in children as early as possible, and prevent its risk factors, so as to avoid the adverse effects of precocious puberty on children. Based on this, this study will explore the relevant influencing factors of precocious puberty in children.

## Data and methods

### Clinical data

The purpose of this study was to explore the related factors of precocious puberty in children. Method analysis 1239 children who underwent physical examination in our hospital from January 2020 to December 2022 were analyzed, including 198 precocious children and 1041 normal children. According to the age of 198 precocious children and 1041 normal children, 205 normal children were selected, and the remaining 836 normal children were excluded. They were divided into precocious group and normal group. Diagnostic criteria for children with precocious puberty: according to the diagnostic criteria for precocious puberty in Pediatrics [8], girls above 8 years old and boys before 10 years old have secondary sexual signs. Girls can be diagnosed as precocious puberty if their body shape changes, breast enlargement, axillary hair and pubic hair reproduction, or if the areola becomes larger than 3 cm or menarche occurs at 10 years old; The diagnosis can be made when a boy over 9 years old has an enlarged laryngeal node, a beard or pubic hair, and testicular development of more than 4 mm. Inclusion criteria for children with precocious puberty: those who meet the above diagnostic criteria; The child’s mental state is normal. Inclusion criteria for normal children: all were examined in our hospital, and their age matched that of precocious children; those who do not meet the diagnostic criteria for sexual precocity; those who have no other diseases with abnormal growth and development; Parents have the willingness and ability to participate in the questionnaire. Exclusion criteria: those with heart disease, liver and kidney; Patients with tumor; those with endocrine or metabolic diseases; those who are combined with infectious diseases such as tuberculosis; Children with precocious puberty who have clear causes such as taking contraceptives and breast cream by mistake; those who combined with organic basic diseases of central nervous system, congenital hypothyroidism, congenital adrenal hyperplasia, etc. This research protocol complies with the relevant requirements of the Helsinki Declaration of the World Medical Association. The study protocol was approved by the Ethics Committee of The First Hospital of Hebei Medical University. Informed consent was obtained from their parents and/or legal guardian before enrollment.

### Method

All medical and nursing personnel in this study have been trained and professionally examined before taking up their posts. All the data of the subjects and parents are confidential and have signed privacy and confidentiality clauses. It collects age, sex, child height, body mass, waist circumference, secondary sexual characteristics according to Tanner’s staging criteria [9], living environment, diet and sleep time; At the same time, the educational background, height, mother’s menarche time and childbearing age of the parents were recorded.

Tanner stage of girls: breast stage: stage I: infant type, with only slight nipple bulge; Stage II: in the bud stage, the breast and nipple bulge like a small mound, with an increase in the diameter of the areola; Stage III: the breast and areola were further enlarged, the side was semicircular, and the areola pigment was increased; Stage IV: the breast is further enlarged, the areola and the breast entering the flesh are enlarged, and the lateral projection is on the semicircle of the breast; Stage V: adult type, breast shape is similar to adult breast, and the second dome of areola disappears; Stage of pubic hair: stage I: no pubic hair; Stage II: The labium majus pudendi has a little thin and light black hair at first; Stage III: The color of the medical dispute deepened and thickened, and began to curl, extending to the pubic symphysis; Stage IV: pubic hair increased significantly, and its pigment, thickness and length were close to those of adults, but limited to the pubic caruncle; Stage V: It is in inverted triangle distribution like the Chinese character of human and spreads to the lower abdomen and thigh root.

Tanner stage of boys: external genitalia: stage I: juvenile type, the shape of penis, testis and scrotum has no obvious change, the diameter of testis is less than 2.5 cm (1–3 ml); Stage II: bilateral testicles and scrotum slightly enlarged, scrotal skin became red, thin and wrinkled, penis slightly enlarged, testicular diameter > 2.5 cm (4–8 ml); Stage III: the double testicles and scrotum continue to increase, the penis increases and thickens, and the testicular diameter is more than 3.5 cm (10-15ml); Stage IV: The penis grows and thickens significantly, the glans penis is exposed, the scrotal skin becomes darker, and the testicle diameter is about 4 cm (15–20 ml); Stage V: adult type, testicular diameter > 4 cm (> 20ml). Pubic hair: stage I: no pubic hair; Stage II: the distribution is limited to the root of the penis, and the fine pubic hair is light; Stage III: pubic hair increased significantly and thickened, distributed in the thigh and extended to the pubic bone; Stage V: adult type, pubic hair is distributed in the midline of the abdomen in a rhombus shape from the bottom to the outside to the inside of the thigh.

Serological indicators: 5 mL of fasting venous blood from all the subjects was taken, centrifuged and sent for examination. The estradiol (E2, the kit was purchased from Wuhan Boster Biological Technology Co., Ltd., batch number: CK-E30580R), follicle-stimulating hormone (FSH, the kit was purchased from Jiangsu Enzyme-Biao Biotechnology Co., Ltd., batch number: MB-6623 A), luteinizing hormone (LH, the kit was purchased from Jiangsu Enzyme-Biao Biotechnology Co., Ltd., batch number: MB-216 A), level of Leptin (ProSpec-Tany, Israel, batch number: CYT-683).

Determination of bone age: the height and body mass index were measured by the same endocrinologist, and measured according to the G-P atlas method, and evaluated by more than two specialists. If the error is more than 6 months, the third specialist should participate.

### Observation indicators

The general data of the two groups were recorded. Logistic regression was used to analyze the influencing factors of precocious puberty in children.

### Statistical analysis

SPSS 21.0 software was used to analyze the data and Excel was used to establish the database. The measurement data conforming to the normal distribution is expressed in $\overline x$ s, the overall comparison of the data of each group is performed by one-way ANOVA, and the pairwise comparison of the data between groups and within groups is performed by LSD method; The counting data is expressed in percentage (%), chi-square χ 2 is used to check and compare; Single and multiple factor analysis Logistic regression was used to analyze the influencing factors of precocious puberty in children. The difference was statistically significant with P < 0.05.

## Results

### Comparison of general data of each group

There was no significant difference between the two groups in children’s height, body mass, birth weight, gestational age, birth order, birth mode, use of adult skin care/cosmetics, father’s height and mother’s height (P > 0.05); The difference was not statistically significant (P < 0.05); There were statistically significant differences between the two groups (P < 0.05) in sex, bone age, daily exercise time, E2, FSH, LH, leptin, mother’s menarche time, living environment, consumption of nutritional supplements, consumption of foods containing pigments and preservatives, consumption of high-protein foods, and sleeping time, as shown in Table 1.

**Table 1:** Comparison of general data of each group

Item	Precocious group (n = 198)	Normal group (n = 205)	χ^2^/t	P
Gender (male/female) (n)	11/187	14/191	0.281	0.596
Age (years)	8.76 ± 1.87	8.69 ± 1.93	0.371	0.712
Child height (cm)	134.38 ± 23.39	129.76 ± 26.56	1.752	0.081
Body mass index (kg/m^2^)	17.56 ± 2.13	17.28 ± 2.09	1.332	0.184
Birth status				
Birth weight (g)	3286.39 ± 129.18	3291.28 ± 135.98	-0.371	0.712
Birth gestational age (W)	38.85 ± 2.29	38.91 ± 2.48	-0.252	0.801
Birth order (times)	1.43 ± 0.32	1.37 ± 0.36	1.766	0.078
Parity (times)	1.17 ± 0.28	1.16 ± 0.36	0.311	0.755
Birth mode (n)			0.247	0.619
Natural labor	85	83		
Cesarean section	113	122		
Bone age (years)	10.38 ± 2.31	7.65 ± 2.18	12.205	＜0.001
Daily exercise time	0.81 ± 0.12	1.32 ± 0.32	-21.042	＜0.001
Serum factor				
E2 (pmol/L)	66.39 ± 13.27	28.28 ± 3.29	39.872	＜0.001
FSH (U/L)	6.01 ± 1.28	2.45 ± 0.76	34.085	＜0.001
LH (U/L)	3.56 ± 0.67	1.12 ± 0.31	47.177	＜0.001
Leptin (µg/L)	8.13 ± 1.34	3.07 ± 1.01	42.902	＜0.001
Use of adult skin care/cosmetics (n)	12	9	0.551	0.458
Father’s height (cm)	173.29 ± 13.29	172.98 ± 12.31	0.243	0.808
Mother’s height (cm)	160.45 ± 12.13	162.32 ± 11.23	-1.607	0.109
Mother’s menarche time (n, < 12 years old)	67	11	51.919	＜0.001
Maternal birth age (n, years)	26.33 ± 5.73	27.41 ± 5.69	-1.898	＜0.058
Parents with college education or above (n, both of them)	27	41	2.908	0.088
Living environment (n, chemical industry zone)	28	4	20.475	＜0.001
Consumption of nutritional supplements (n, often)	127	32	99.309	＜0.001
Consumption of food containing pigments and preservatives (n, often)	118	41	66.106	＜0.001
Consumption of high-fat foods (n, often)	135	38	101.322	＜0.001
Sleep time (h)	9.03 ± 1.57	11.08 ± 1.34	-14.115	＜0.001

### Single factor logistic regression analysis of factors affecting precocious puberty in children

The independent variables were set as sex (female), bone age (> 10 years old), daily exercise time (< 0.9 h), E2 (≥ 66.00pmol/L), FSH (≥ 6.00U/L), LH (≥ 3.50U/L), leptin (≥ 8.00 µ G/L), mother’s menarche time (< 12 years old), living environment (chemical industry zone), consumption of nutritional supplements (often), consumption of foods containing pigments and preservatives (often), consumption of high-protein foods (often), and sleep time (< 10 h). The dependent variables are children’s precocious puberty. The single factor Logistic regression analysis shows that the risk factors of children’s precocious puberty are gender (female), bone age (> 10 years old) Daily exercise time (< 0.9 h), E2 (≥ 66.00pmol/L), FSH (≥ 6.00U/L), LH (≥ 3.50U/L), leptin (≥ 8.00 µ G/L), mother’s menarche time (< 12 years old), living environment (chemical industry zone), consumption of nutritional supplements (often), consumption of high-protein food (often), and sleep time (< 10 h), as shown in Table 2.

**Table 2:** Single factor Logistic regression analysis of factors affecting precocious puberty in children

Variable	β	SE	Wdlodχ^2^ value	OR (95%CI)	P value
Gender (female)	1.263	0.308	14.591	3.018 (1.895, 7.105)	0.000
Bone age (> 10 years old)	1.451	0.307	21.376	4.067 (1.281, 7.587)	＜0.001
Daily exercise time (< 0.9 h)	0.811	0.309	7.517	2.321 (1.165, 3.765)	0.006
E2 (≥ 66.00pmol/L)	1.229	0.328	12.062	3.167 (1.317, 6.887)	＜0.000
FSH (≥ 6.00U/L)	0.928	0.289	10.165	2.537 (1.437, 4.779)	＜0.001
LH (≥ 3.50U/L)	0.867	0.157	29.876	2.287 (1.652–3.281)	＜0.001
Leptin (≥ 8.00 µg/L)	0.367	0.139	6.065	1.453 (1.092–1.786)	0.008
Mother’s menarche time (< 12 years old)	0.889	0.183	23.287	0.761 (0.327–0.998)	＜0.001
Living environment (chemical industry zone)	1.772	0.376	22.387	4.382 (1.891–10.987)	＜0.001
Consumption of nutritional supplements (n, often)	0.276	0.113	6.038	1.287 (1.046–1.578)	0.009
Consumption of food containing pigments and preservatives (n, often)	0.028	0.037	0.947	1.028 (0.976, 1.128)	0.318
Consumption of high-fat foods (n, often)	2.298	1.098	4.218	8.912 (1.038–7.398)	0.031
Sleep time (< 10 h)	0.709	1.019	0.482	2.029 (0.268–9.876)	0.476

### Multi-factor logistic regression analysis of factors affecting precocious puberty in children

The independent variables were set as sex (female), bone age (> 10 years old), daily exercise time (< 0.9 h), E2 (≥ 66.00pmol/L), FSH (≥ 6.00U/L), LH (≥ 3.50U/L), leptin (≥ 8.00 µ G/L), mother’s menarche time (< 12 years old), living environment (chemical industry zone), consumption of nutritional supplements (often), consumption of high-protein food (often), and sleep time (< 10 h). The dependent variables are children’s precocious puberty. Multifactor logistic regression analysis shows that the risk factors of children’s precocious puberty are gender (female), bone age (> 10 years old), and daily exercise time (< 0.9 h) E2 (≥ 66.00pmol/L), FSH (≥ 6.00U/L), LH (≥ 3.50U/L), leptin (≥ 8.00 µ G/L), mother’s menarche time (< 12 years old), living environment (chemical industry zone), consumption of nutritional supplements (often), consumption of high-protein food (often), sleep time (< 10 h), as shown in Table 3.

**Table 3:** Multi-factor Logistic regression analysis of factors affecting precocious puberty in children

Variable	β	SE	Wdlodχ^2^ value	OR (95%CI)	P value
Gender (female)	0.438	0.159	2.387	1.476 (1.139–2.009)	0.005
Bone age (> 10 years old)	1.003	0.159	6.382	2.387 (1.021–3.987)	＜0.001
Daily exercise time (< 0.9 h)	1.327	0.247	4.382	3.287 (2.381–4.398)	＜0.001
E2 (≥ 66.00pmol/L)	1.019	0.382	2.673	2.671 (1.238–4.382)	0.006
FSH (≥ 6.00U/L)	1.769	0.332	4.387	4.392 (2.391–7.913)	＜0.001
LH (≥ 3.50U/L)	2.187	0.278	6.387	7.761 (3.193–8.887)	＜0.001
Leptin (≥ 8.00 µg/L)	0.971	0.439	5.021	2.761 (1.109–4.302)	0.019
Mother’s menarche time (< 12 years old)	1.637	0.635	6.038	5.238 (1.309–8.391)	0.012
Family history (Yes)	1.728	0.609	7.382	5.781 (1.387–12.109)	0.004
Living environment (chemical industry zone)	1.582	0.632	4.321	4.871 (1.328–17.398)	0.021
Consumption of nutritional supplements (n, often)	2.239	0.621	12.398	6.398 (2.013–9.871)	＜0.001
Consumption of high-fat foods (n, often)	0.387	0.183	2.165	1.437 (1.038–2.078)	0.029
Sleep time (< 10 h)	0.023	0.003	3.273	1.023 (1.002–1.439)	0.001

## Discussion

With the improvement of living standards, the problem of children’s precocious puberty is becoming increasingly serious, and parents and doctors are paying more and more attention to it, because children’s precocious puberty will bring lots of harm to children; In terms of physiology and pathology, the height, weight and bone age of children with precocious puberty all develop rapidly. Precocious puberty will lead to early bone age development of children, early closure of epiphysis, and shortened growth period. The final height of adults will be affected, and serious cases will lead to dwarfism; Precocious puberty can lead to secondary sexual symptoms in children, even menstrual cramps; Premature puberty also increases the risk of cancer [10, 11]. Foreign studies have pointed out that girls with precocious puberty will increase the risk of breast cancer and uterine cancer; In addition, from the perspective of social psychology, changes in mentality caused by sexual development will increase the psychological burden of children, and even cause social problems such as early sexual behavior of children or juvenile sexual crimes [12]. Domestic research points out that the age of children’s precocious puberty has a tendency of appearing in children at younger age. Among them, idiopathic central precocious puberty is the most common, accounting for 92% of precocious puberty. Children’s precocious puberty has become the second major problem that plagues children’s healthy growth [13]. Therefore, the prevention and treatment of precocious puberty in children has become a hot issue in clinical research at this stage. Therefore, it is necessary to analyze its influencing factors to better prevent the occurrence of precocious puberty.

Yan Xuemei [14] et al. pointed out that frequent consumption of nutritious foods and mother’s menarche age ≤ 13 years old are independent risk factors affecting children’s precocious puberty. Yi Haiqun [15] et al. pointed out that LH, FSH, E2, diet and living habits are independent risk factors affecting precocious puberty in children. Liu Lifang [16] and other researchers pointed out that children’s growth and development are affected by various factors. Strengthening children’s health care can help ensure children’s normal development, thus promoting children’s physical and mental health. The results of this study showed that comparison between the two groups in the sex, bone age, daily exercise time, E2, FSH, LH, leptin, mother’s menarche time, family history, living environment, consumption of nutritional supplements, consumption of food containing pigments and preservatives, consumption of high-protein food, work and rest (P < 0.05), suggest that children’s precocious puberty may be related to sex, bone age, daily exercise time, E2, FSH, LH, leptin, mother’s menarche time, family history, living environment, consumption of nutritional supplements, food with pigments and preservatives, food with high protein, work and rest.

The current research has made it clear [17] that there are significant differences between girls and boys in endocrine and growth and development, and studies have confirmed that the incidence of precocious puberty in girls is significantly higher than that in boys. Bone age is a common detection index for bone examination, which refers to the relationship between the appearance and healing time of skeletal bone nucleus and the actual age [18]. Less exercise time per day will lead to a large amount of fat stored and accumulated in the body, stimulating hormone secretion, thus causing sexual precocity [19]. E2 is mainly formed by placenta, corpus luteum and ovarian follicles of pregnant women, and is an important indicator of whether the function and level of sexual hormones are normal [20]. FSH and LH play a synergistic role in maintaining the menstrual cycle, and LH acts on the ovary to synthesize estrogen, and estrogen acts on the growth plate, resulting in accelerated growth, bone maturity, and bone age advance [20]. Leptin is a pleiotropic protein hormone secreted by white adipose tissue, which can regulate the secretion of gonadotropin-releasing hormone, directly stimulate the release of FSH and LH from the pituitary, have direct physiological regulation on ovarian function, promote endometrial thickening and volume increase; In addition, it will affect the fat level of adolescents, lead to the development of puberty, and become the permissive factor for the development of secondary sexual characteristics in the body. When the nutritional status is improved enough to satisfy the development, leptin can affect the hypothalamus-pituitary-gonad axis, affect the level of sex hormones, and start puberty, thus leading to precocious puberty, resulting in premature growth and accelerated bone growth [21]. Under normal circumstances, children’s menarche age will be close to the mother’s menarche age. If the mother has later menarche, their children will also develop later [22]. Some studies have pointed out that mother’s menarche time is closely related to children’s precocious puberty. Contamination of residential areas in industrial areas will cause endocrine imbalance in children and induce precocious puberty [22]. Consumption of nutritional supplements and high-protein foods will lead to excessive energy intake, and the excess energy will be stored in the body in the form of fat, thus affecting the endocrine [23]. A short sleep time will affect the normal work of the pineal gland, the secretory organ in the brain, thus inducing sexual precocity [24]. Gong Dai [25] et al. pointed out that children can increase the risk of precocious puberty due to eating contaminated food, high leptin level and short daily exercise time. Wu Chuting [26] et al. pointed out that the occurrence of precocious puberty in children is related to living habits, eating habits, mother’s age at menarche, E2, FSH, LH, leptin and other factors. The results of this study showed that the risk factors of precocious puberty in children were sex (female), bone age (> 10 years old), daily exercise time (< 0.9 h), E2 (≥ 66.00pmol/L), FSH (≥ 6.00U/L), LH (≥ 3.50U/L), leptin (≥ 8.00 µ G/L), mother’s menarche time (< 12 years old), living environment (chemical industry zone), consumption of nutritional supplements (often), consumption of high-protein food (often), sleep time (< 10 h). For further study, multivariate logistic regression analysis was used to analyze the influencing factors of precocious puberty in children. The results showed that the risk factors of precocious puberty in children were gender (female), bone age (> 10 years old), daily exercise time (< 0.9 h), E2 (≥ 66.00pmol/L), FSH (≥ 6.00U/L), LH (≥ 3.50U/L), leptin (≥ 8.00 µ G/L), mother’s menarche time (< 12 years old), living environment (chemical industry zone), consumption of nutritional supplements (often), consumption of high-protein food (often), and sleep time (< 10 h).

Therefore, clinicians should pay attention to children’s gender, bone age, exercise habits, E2, FSH, LH, leptin, mother’s menarche time, living environment, eating habits, sleep time and other factors to alert the occurrence of precocious puberty. It is important to detect bone age, E2, FSH, LH and leptin levels in children every year. Parents should reasonably arrange children’s eating habits and exercise habits, and avoid high-fat diets to reduce excessive accumulation of fat. In addition, a suitable living environment can also help prevent the occurrence of premature puberty in children. There are still some shortcomings in this study. First, this study is a retrospective observational study with a small sample size, which may weaken the generalisability of the results. Second, some characteristic data spans too long and may be inexact, which may introduce bias. Third, some precocious puberty symptoms are not obvious, which may lead to selection bias. In the next study, we will conduct a prospective study with a large sample size to explore this topic.

## Conclusion

In conclusion, children’s gender, bone age, exercise habits, E2, FSH, LH, leptin, mother’s menarche time, living environment, eating habits, sleep time and other factors are closely related to precocious puberty in children. Reminding parents to actively prevent related factors in clinical work is helpful to prevent the occurrence of precocious puberty in children.

## Data Availability

The datasets generated and analyzed during the current study are available from the corresponding author on reasonable request.
