# Correction: Analysis of risk factors of precocious puberty in children

**DOI:** 10.1186/s12887-023-04320-7

**Published:** 2023-09-22

**Authors:** Yan Dong, Lili Dai, Yang Dong, Na Wang, Jing Zhang, Chao Liu, Zhifang Li, Limin Chu, Sisi Chen

**Affiliations:** 1 Department of Pediatrics, The First Hospital of Hebei Medical University, No. 89 Donggang Road, Yuhua District, Shijiazhuang, Hebei 050000 China; 2 Department of Laboratory Medicine, The First Hospital of Hebei Medical University, Shijiazhuang, China; 3 Department of Laboratory Medicine, The Third Hospital of Shijiazhuang, Shijiazhuang, China

**Correction:** ***BMC Pediatr*** **23, 456 (2023)**

10.1186/s12887-023-04265-x

Following publication of the original article [1], the authors would like to correct the corresponding author’s name from “Sisi Chen” to “Sisi Cheng”.
